# Application of a Knowledge, Attitude, Belief, and Practice Model in Pain Management of Patients with Acute Traumatic Fractures and Alcohol Dependence

**DOI:** 10.1155/2022/8110896

**Published:** 2022-02-15

**Authors:** Ying Dong, Hui Gao, Zheyu Jin, Jue Zhu, Hao Yu, Yingqing Jiang, Jun Zou

**Affiliations:** Department of Orthopaedic Surgery, The First Affiliated Hospital of Soochow University, Suzhou, Jiangsu 215006, China

## Abstract

**Objective:**

To evaluate the outcome of a knowledge, attitude, belief, and practice mode (KABP) in the pain management in patients with acute traumatic fractures complicated with alcohol dependence.

**Methods:**

Twenty-nine alcohol-dependent male patients with acute traumatic fractures and who received surgical treatment between January 2019 and December 2020 were included in this retrospective case-control study. The age range was 30–65 years (average 50.03 ± 7.94). *Fracture Type*. Six cases of spinal burst fractures and 23 cases of limb trauma fractures. Ten patients were treated with routine nursing (control group), and 19 patients were treated with pain management in KABP mode (experimental group). The control group received traditional pain care, including the conventional numerical rating scale (NRS) pain score system, with focus on symptomatic treatment. On this basis, the experimental group managed pain using KABP, including cognitive behavioral intervention, optimization programs, modification of personal beliefs, and behavior patterns. NRS, self-rated anxiety/depression scale (SAS), and quality of life (SF-36) scale were applied at admission, 1 day before surgery, and 3 months after surgery.

**Results:**

The perioperative NRS score of the KABP group was lower than that of the control group, and the postoperative anxiety levels improved. Discharge satisfaction was significantly higher than that in the control group (*p* < 0.05). There were behaviors promoting health in the experimental group, and five patients expressed abstinence behavior after discharge (*p* < 0.05).

**Conclusion:**

Patients with alcohol dependence represents a unique set of cases for perioperative pain management. To ensure patient safety, individualized pain management through the application of KABP can significantly reduce postoperative pain and promote the generation of healthy behaviors in patients.

## 1. Introduction

Perception and knowledge of pain increased substantially in the past two decades. In August 2002, at the International Pain Society (IASP)'s 10th World Congress on Pain in San Diego, California, USA, a consensus was reached that pain is the fifth vital sign, after blood pressure, body temperature, respiration, and pulse [1]. Alcohol is a psychoactive substance with toxic and addictive properties and fast distribution after absorption. Alcohol dependence is also commonly known as “alcohol addiction.” A large number of patients with alcohol dependence on long-term drinking may present slow response, poor concentration and memory, and may seriously impair the nervous system [2]. Patients with alcohol dependence and acute trauma fractures, due to sudden interruption of alcohol stimulation after admission, demonstrate withdrawal symptoms [3].

Significant postoperative pain is often experienced in trauma-related orthopedic surgeries. Patients with alcohol dependence often experience greater pain, compared to the ordinary population [4], as conventional analgesic measures are often ineffective. Such patients often resist surgery, due to alcohol withdrawal symptoms or fear of significant postoperative pain. This often results in a series of physical and psychological problems. Therefore, individualized pain control and management are essential for patients with acute traumatic fractures and alcohol dependence. There are no clinical pain management protocols for patients with alcohol dependence. Behavioral studies [5] show that there is a strong association between knowledge and behavior, but it is not entirely causal. A person's behavior is multifactorial, being related to knowledge, values, beliefs, living environment, and personal experience [6]. The knowledge, attitude, belief, and practice mode (KABP) hold that knowledge and information are the basis for establishing positive and correct beliefs and attitudes, thus promoting health-related behaviors, while beliefs and attitudes are the driving forces for behavior change [7]. To this end, we conducted a retrospective case-control study to evaluate the outcomes of KABP and traditional nursing modes in the perioperative pain management of patients with acute traumatic fractures complicated by alcohol dependence.

## 2. Materials and Methods

Inclusion criteria were as follows: (1) patients with alcohol dependence and demonstration of alcoholic withdrawal syndromes; (2) no history of compulsory abstinence therapy prior to admission; (3) no signs of delirium and cognitive impairment. The exclusion criteria were as follows: (1) patients with Alzheimer's disease, disturbance of consciousness, mental illness, and poor cooperation; (2) malignant tumor and severe hepatic and renal failure.

A total of 29 patients with acute traumatic fractures complicated by alcohol dependence were included, all of whom were men. The age ranged from 30 to 65 years (50.03 ± 7.94) years. Fracture type: six cases of spine burst fracture and 23 cases of limb trauma fracture. Ten patients were treated with conventional nursing (control group), and 19 patients were treated with pain management in the KABP mode (experimental group). There was no statistically significant difference in baseline data between the two groups (*P* > 0.05), indicating a good comparability (Table 1). Written informed consent was obtained from all patients. This study was approved by the Medical Ethics Committee of the First Affiliated Hospital of Soochow University.

### 2.1. Nursing Methods

#### 2.1.1. Control Group

The routine orthopedic pain management mode was used. Upon admission, the bed nurse performed pain assessment using the NRS pain assessment scale. Analgesic treatments were performed according to the evaluation results. A stepwise analgesic drug use principle was adopted. All patients received general symptomatic treatment.

#### 2.1.2. The Experimental Group

**Figure 1:**
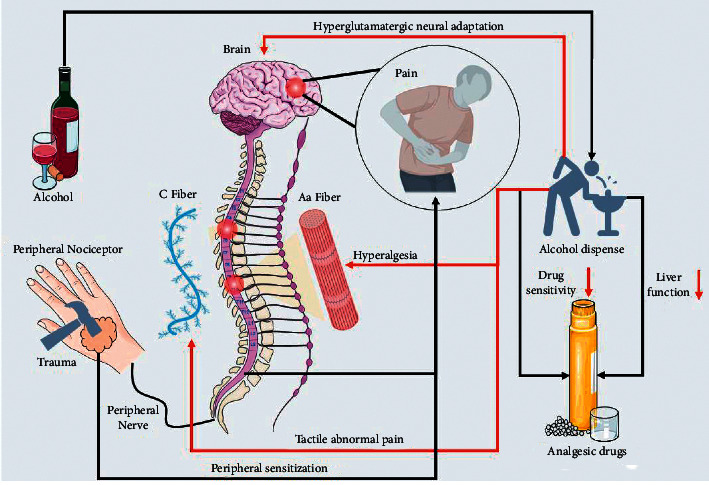
Alcohol and pain mechanisms.

On the basis of conventional nursing, KABP mode was applied for all patients, including the following:

(1) Cognitive intervention: cognitive intervention is an effective measure to improve the quality of pain management and occupies an important position in the perioperative nursing of orthopedic surgery [8]. Preoperatively, medical staff should provide patients with a general understanding of the surgical procedure and timely communication with the patient, so that the patient can change the initial cognition of perioperative pain as well as correct cognition of the operation. The influence of alcohol on perioperative pain is shown in Figure 1. Therefore, KABP mode reduces the risk of postoperative complications and enhances patient recovery.

Cognitive intervention for alcohol withdrawal syndromes: Alcohol withdrawal syndrome mainly includes mental and somatic symptoms. Somatic symptoms generally occur within 7–48 hours after reducing alcohol intake or complete withdrawal, mainly demonstrating symptoms of tremor, sweating, tachycardia, elevated blood pressure, and other aspects, while the threshold of pain is reduced [9]. Psychiatric symptoms usually occur 48–72 hours after alcohol consumption is reduced, or withdrawn, and are characterized by severe confusion, loss of orientation, vivid daydream-like delusions and hallucinations, accompanied by anxiety, insomnia, and hypersympathetic activity. Cognitive intervention measures at this stage mainly include knowledge education and targeted treatment, explaining the withdrawal symptoms to patients, alleviating the occurrence of withdrawal symptoms, and reducing their anxiety and fear through rational use of drugs. Commonly used drugs include benzodiazepines, vitamins, and naloxone [10].

(2) Changes in beliefs and attitudes: beliefs come from four factors: knowledge, environment, happenstance, and successful experience [11]. Optimization of postoperative pain management is the main method to reduce patients' fear of surgery and refusal of treatment. Alcohol-dependent patients have reduced thresholds to pain and sensitivity to pain medications. Intense pain experience will bring detrimental emotions to patients, which will affect their attitude toward intervention, or even reject the implementation of intervention measures. For such patients, the optimization strategy recommended the principles of multimodal analgesia, advanced analgesia, and individualized analgesia (medication on demand instead of on-time).

The 2012 American Society of Anesthesiologists recommended that multiple modes of analgesia should be used as much as possible [12]. Multimode analgesia refers to the combined use of analgesic drugs. It also involves the use of different methods or mechanisms of action to achieve additive or synergistic effects without increasing complications. Simultaneously, the dosage of each drug is steadily reduced, as are the adverse reactions. The objective is to achieve the maximum balanced analgesic effect that is conducive to patients with surgical pain.

Advanced analgesia: the objective of preventive analgesia is to reduce or eliminate sensitization that is caused by harmful stimuli in the perioperative period. Preventive analgesia can inhibit peripheral and central sensitization, reduce postoperative pain intensity, and reduce the demand for analgesics [13].

Medications: medications that rapidly cross the blood-brain barrier to inhibit central sensitization, including selective COX-2 inhibitors.

Individualized analgesia: on-demand symptomatic treatment is transformed into on-time and on-volume analgesia treatment [14]. The treatment plan mainly includes the following: after 1-2 days of parecoxib sodium alone after mild pain, oral Celebrex 200 mg bid 5–7 days after PCA after moderate pain, combined with parecoxib sodium 2-3 days, oral Celebrex 200 mg bid for 5–7 days. If PCA is not used, tramadol plus parecoxib should be used for 2-3 days. PCA was used after severe pain surgery, and Celebrex 200 mg bid for 5–7 days after the combined use of parecoxib sodium for 3–7 days. A combination of peripheral nerve block, plexus block, and sustained-release opioid analgesics was used when necessary.

(3) Behavioral interventions: by increasing the cognitive behavior intervention, an optimized scheme to set up faith to change the behavior of patients was sought [15]. The experimental group was encouraged to participate in pain management through the intervention of pain and alcohol withdrawal cognition during the perioperative period, and self-pain score was performed. The analgesic program should be optimized to increase patients' confidence in pain control through successful analgesic experience or their own experience so that patients can carry out healthy behaviors.

On the premise of alleviating pain in patients, optimization of rehabilitation programs for patients. Functional exercise was performed under pain control, supplemented by dietary education, to improve the comfort of patients and enhance recovery.

### 2.2. Observation Indicators

(1) The NRS digital scoring scale was used to compare the pain scores of patients in the two groups on admission, 1 day before surgery, 1 day after surgery, and 3 months after surgery. (2) SAS anxiety/depression scale was used to compare the anxiety curve of the two groups at admission, 1 day before surgery, and 3 months after surgery. (3) The changes in health behaviors in the two groups were compared using the quality of life scale. (4) Nursing satisfaction was compared between the two groups. When the patients were discharged, a satisfaction questionnaire was issued. It included the following information: satisfaction with the intervention measures, functional exercise guidance, health education, and other contents, with a total score of 100 points, 80–100 points were very satisfied, 60–79 points were satisfied, and <60 points were not satisfied. Overall satisfaction was calculated as follows: (very satisfied + satisfied)/total number of cases × 100%.

### 2.3. Statistical Analysis

The application of SPSS 23.0, the Shapiro–Wilk method to normality test data, accorded with normal distribution, according to measurement data to mean + SD group comparison between the two independent samples *t*-tests; measurement data that did not conform to normal distribution were represented by *M* (Q1, Q3), and the rank sum test was used for comparison of groups. Statistical data were expressed as percentages and the *χ*^2^ test was used for comparison between groups. Statistical significance was set at *p* < 0.05.

## 3. Results

### 3.1. The NRS Scoring Scale

The NRS scoring scale was used at 1 day preoperatively, on the day of operation, 1 day, and 3 months postoperatively to compare between the control group and the experimental group. The results are given in Table 2.

There was no statistically significant difference between the two groups in pain score at admission and 1 day preoperatively. Significant differences were observed on the day of surgery and postoperatively. The NRS pain.

Scores were significantly lower than the control group, indicating that the KABP intervention is effective and can significantly reduce the patient's pain.

### 3.2. The SAS Anxiety/Depression Scale

**Figure 2:**
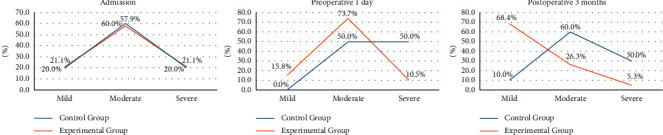
Comparison of patient anxiety.

The SAS anxiety/depression scale was used to compare the anxiety curve of the two groups on admission, 1 day before surgery, and 3 months after surgery. The results are given in Table 3 and Figure 2.

No statistical difference in psychological anxiety at admission was observed between the experimental and control groups. The application of cognitive intervention showed that anxiety level was significantly alleviated in the experimental group, compared to the control group 1 day before and 3 months after surgery (*p* < 0.05).

### 3.3. The SF-36 Health Status Questionnaire

The SF-36 health status questionnaire was used to evaluate the patients' quality of life from eight aspects, including physical function, physical role, physical pain, general health, vitality, social function, emotional function, and mental health. The quality of life assessment scale (SF-36) was used at admission and 3 months after surgery to compare the differences in quality of life between the two groups. The results are given in Tables 4 and 5.

The results showed that the scores of the two groups at admission were not statistically significant. However, due to fracture trauma, the quality of the patients' life in both groups decreased to varying degrees within three months of surgery. Comparatively speaking, the scores of the control group were lower, and the results were statistically significant. In comparison within the same group, the quality of life of the control group was significantly reduced, and the results were significant (*p* < 0.05). No significant decrease in SF-36 scores was observed in the experimental group (*p* < 0.05). In addition, in this study, there were five patients in the experimental group who took the initiative to express abstinence determination and/or action.

### 3.4. Comparison of Nursing Satisfaction

Comparison of nursing satisfaction between the two groups is given in Table 6.

The satisfaction of the experimental group was significantly higher than that of the control group, and the results were statistically significant (*p* < 0.05).

## 4. Discussion

The sharp increase of alcohol consumption in China since the 1980s and a series of social and economic problems were caused by excessive drinking [16]. Alcohol abuse is the fifth major risk factor for premature death and disability globally and is the leading cause of death and disability in developing countries. The WHO Global Action Plan for the Prevention and Control of Noncommunicable Diseases (2013–2020) formulated that the voluntary global target was to reach a 10% reduction in the harmful use of alcohol, as appropriate, within the national context [17]. However, through numerous literature reviews, the authors found that there is still a lack of large-scale national epidemiological investigations on the Chinese alcoholic population, and there are only investigations and studies on the organic damage caused by alcohol dependence, such as alcoholic hepatitis in foreign countries [18]. The foundation of alcohol control is weak, and a complete working system has not yet been formed.

### 4.1. Pain Management

Pain management remains a critical public health issue worldwide, particularly for orthopedic surgery patients. Patients with acute traumatic fractures and alcohol dependence require specific pain management techniques. However, these patients do not receive sufficient clinical attention, and ordinary conventional analgesic measures are often insufficient for pain relief. They often increase unpleasant or even painful emotional experiences, which leads to patients being depressed and refuse to get treated, thus affecting their rehabilitation. Clinical nursing work urgently needs to adopt safe and effective pain management modes for these patients. In this study, patients in the experimental group correctly understood pain and surgery through early cognitive intervention. Patients were encouraged to participate in pain management and self-pain assessment. Through the analgesia optimization strategy, patients' pain score in the perioperative period was significantly lower than that in the control group.

### 4.2. Knowledge, Attitude, Belief, and Practice Mode (KABP)

The KABP divides the change of human behavior into three continuous processes: knowledge, generating belief, and forming behavior [18]. In clinical trials, the goal of the model is ensure patients' consistent compliance after considering their best interests and ensure the efficiency and quality of the trial. The results of this study indicate that the establishment of beliefs and change of attitudes is essential and directly related to the stability of patient compliance.

### 4.3. The KABP Pattern Is Beneficial to Patient Knowledge Mastery and Cognitive Improvement

Long-term, heavy drinking can lead to mental and physical dependence and cognitive dysfunction. Studies have shown that patients with alcohol dependence have impaired cognitive functions (e.g., memory, visual space, and executive function), whereas their general intelligence and familiar knowledge are relatively intact [19]. In this study, patients in the experimental group displayed improved cognitive level and reduced anxiety and fear through repeated cognitive reinforcement in the KABP mode. Regarding anxiety, between the control and experimental groups, there was no difference at admission, a significant difference before surgery, and a significant difference three months after surgery; this indicates that early and repeated interventions of knowledge, belief, and action modes are conducive to anxiety relief in patients.

### 4.4. The KABP Mode Is Conducive to the Establishment of Healthy Behaviors

Multidirectional communication is conducive to patients' understanding of knowledge and mastery of exercise methods. Under the encouragement and guidance of medical staff, it is easier to transform knowledge, methods, and beliefs into healthy behaviors. In this study, the change in patient anxiety, improvement of their sense of participation, and the degree of treatment cooperation indicate that the health education in the KABP mode make patients more receptive. According to the SF-36 health status scores, the quality of life in the experimental group did not significantly decrease three months after surgery, whereas the quality of life in the control group significantly decreased. In the later investigation, five patients in the experimental group took the initiative to express the willingness or action of abstaining from alcohol, reflecting the intervention's positive effect and promoting the establishment of patients' healthy behaviors.

### 4.5. The KABP Mode Is Conducive to Improving Patient Satisfaction

In this study, 30% of patients in the control group and 84.2% in the experimental group were highly satisfied. The education in the KABP mode takes the needs of patients as the starting point, attaches importance to communication and interaction with patients in the intervention process to significantly improve the degree of attention they feel, and increases patients' satisfaction with nursing work.

In conclusion, on the basis of ensuring safety, individualized pain management programs can significantly reduce patients' pain and promote the generation of healthy behaviors through the application of knowledge, belief, and action mode [20]. This study provides practical guidance for pain management in patients with acute traumatic fractures and alcohol dependence and lays a foundation for the study of surgical pain management in other special populations.

## Figures and Tables

**Table 1:** General information.

Group	Cases	Age	Fracture type		Level of education				Daily alcohol intake (ml)
			All	Spine	College	Junior school	Primary school	Illiteracy	
Control group	10	48.50 ± 9.54	8	2	3	4	1	2	475 ± 118.43
Experimental group	19	50.84 ± 7.12	4	15	8	6	3	2	484 ± 162.49
T/*χ*^2^	—	−0.749	0.004		1.040				−0.158
*P*	—	0.461	0.947		0.791				0.876

**Table 2:** Comparison of pain scores between the two groups (NRS) ($\bar{x}$ ± *s*).

Group	Number of cases	Admission	Preoperative 1 day	Surgery	Postoperative 1 day	Postoperative 3 months
Control group	10	5.10 ± 0.994	4.30 ± 0.675	5.80 ± 1.135	6.10 ± 1.101	4.30 ± 0.823
Experimental group	19	5.00 ± 1.00	3.47 ± 0.612	4.84 ± 1.068	4.95 ± 1.079	2.89 ± 1.197
*t*		0.256	3.339	2.248	2.717	3.310
*P*		0.800	0.002^*∗∗*^	0.033^*∗*^	0.011^*∗*^	0.003^*∗∗*^

**Table 3:** Comparison of patient anxiety.

Group	Number of cases	Admission			Preoperative 1 day			Postoperative 3 months		
		Mild	Moderate	Severe	Mild	Moderate	Severe	Mild	Moderate	Severe
Control group	10	2 (20)	6 (60)	2 (20)	0 (0)	5 (60)	5 (40)	1 (10)	6 (60)	3 (30)
Experimental group	19	4 (21.05)	11 (57.90)	4 (21.05)	3 (10.53)	14 (73.68)	2 (15.79)	13 (68.42)	5 (26.32)	1 (5.26)
*χ* ^2^		0.020			0.424			0.497		
*P*		0.994			0.041^*∗*^			0.009^*∗∗*^		

**Table 4:** Comparison of quality of life scores between the two groups (SF-36).

Group	Number of cases	Admission	Postoperative 3 months
Control group	10	88.20 ± 7.21	77.50 ± 6.33
Experimental group	19	89.74 ± 10.47	88.37 ± 12.42
*t*		−0.414	−2.581
*P*		0.682	0.016

**Table 5:** Comparison of quality of life scores of patients in the same group (SF-36).

Group	Number of cases	Control group	Experimental group
Admission	10	88.20 ± 7.21	89.74 ± 10.47
Postoperative 3 months	19	77.50 ± 6.33	88.37 ± 12.42
*t*		3.527	0.367
*P*		0.002	0.716

**Table 6:** Comparison of satisfaction.

Group	Number of cases	Great satisfaction	Satisfaction	Dissatisfaction
Control group	10	3 (30)	5 (50)	2 (20)
Experimental group	19	16 (84.21)	2 (10.53)	1 (5.26)
*χ* ^2^		8.594		
*P*		0.014		

## Data Availability

The data generated or analyzed during this study are included within the article.
